# Supplementary material for: Intranasal Administration of Codium fragile Polysaccharide Elicits Anti-Cancer Immunity against Lewis Lung Carcinoma
Source: Int J Mol Sci. 2021 Sep 30;22(19):10608. doi: 10.3390/ijms221910608 (PMC8508762; doi:10.3390/ijms221910608)
Supplement: Supplementary file 1 [file ijms-22-10608-s001.zip › ijms-1390359-supplementary.pdf]

Supplementary Materials for

Intranasal administration of *Codium fragile* polysaccharides elicits anti-cancer immunity against Lewis lung carcinoma

Yuhua Wang, Eun-Koung An, Wei Zhang, So-Jung Kim, SangGuan You, and Jun-O Jin

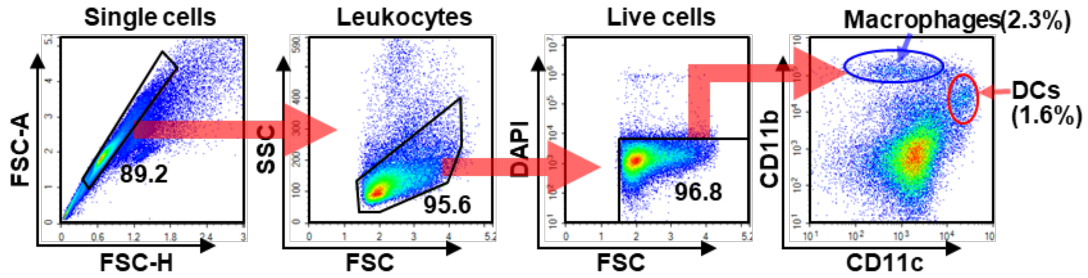

Figure S1. Definition of dendritic cells (DCs) and macrophages in the mediastinal lymph nodes (mLNs).

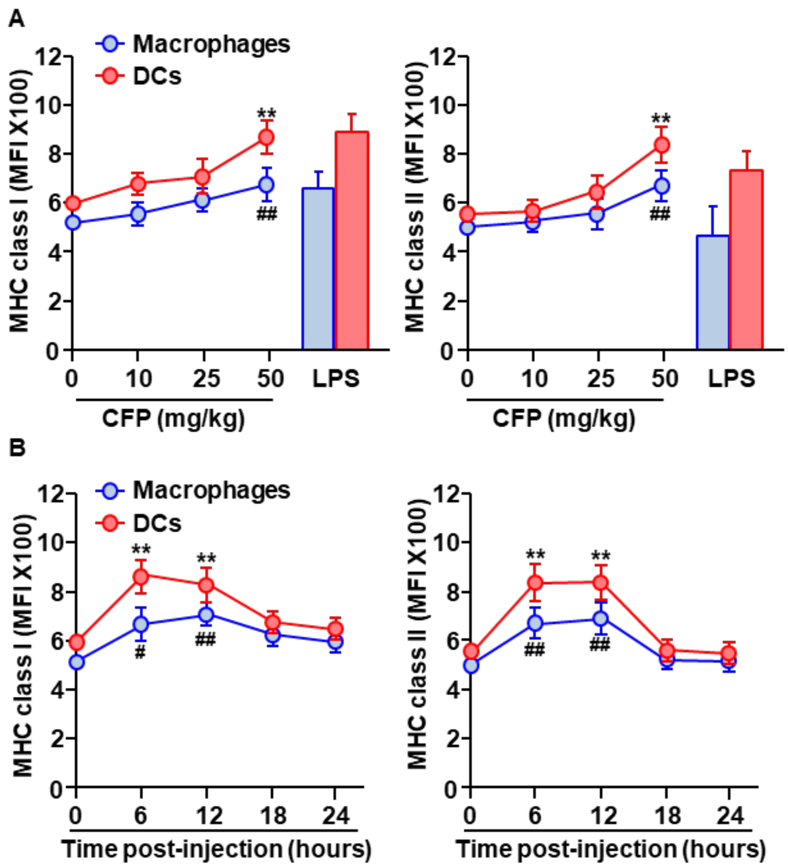

Figure S2. Effect of *Codium fragile* polysaccharides (CFPs) on the activation of DCs and macrophages. C57BL/6 mice were intranasally (*i.n.*) treated with CFPs. Time- and dose-dependent effects on the upregulation of MHC molecules were analyzed in DCs and macrophages.

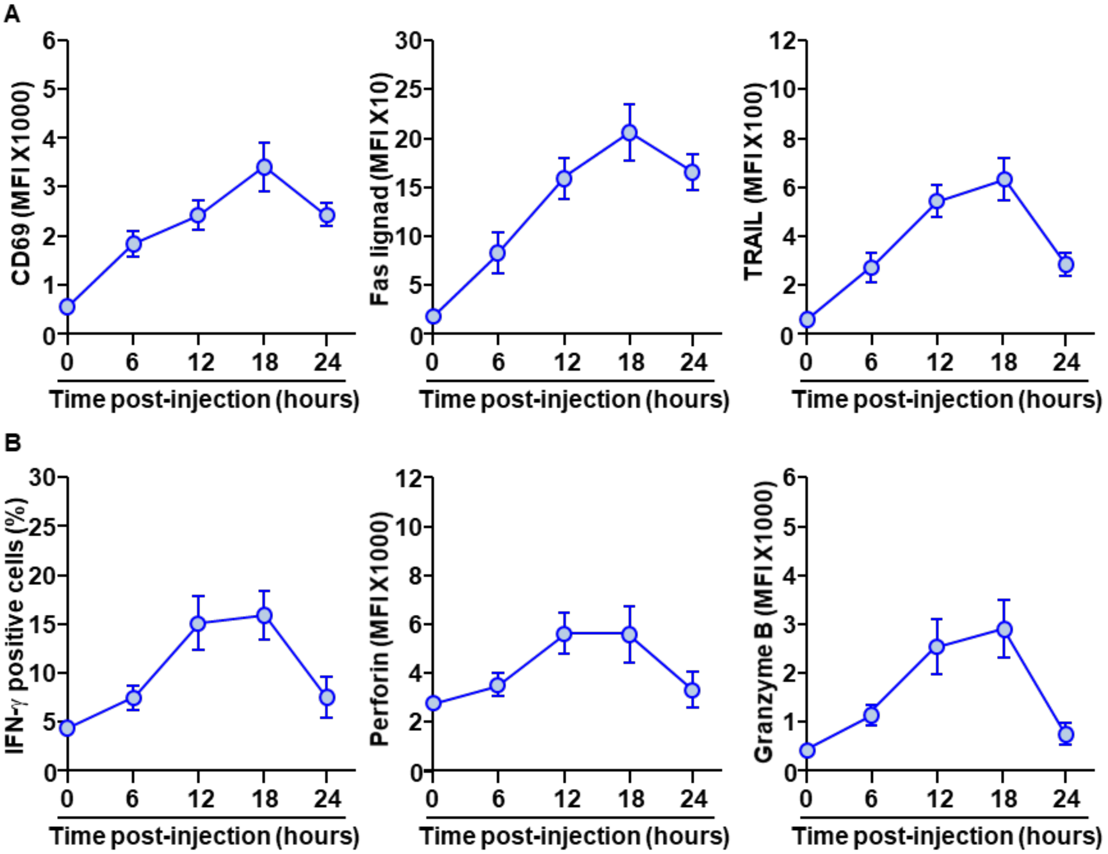

Figure S3. Time dependent effect of CFPs on NK cell activation. The mice were treated as indicated in Figure 3. Intracellular cytokine and cytotoxic mediator levels in mLN NK cells as analyzed.

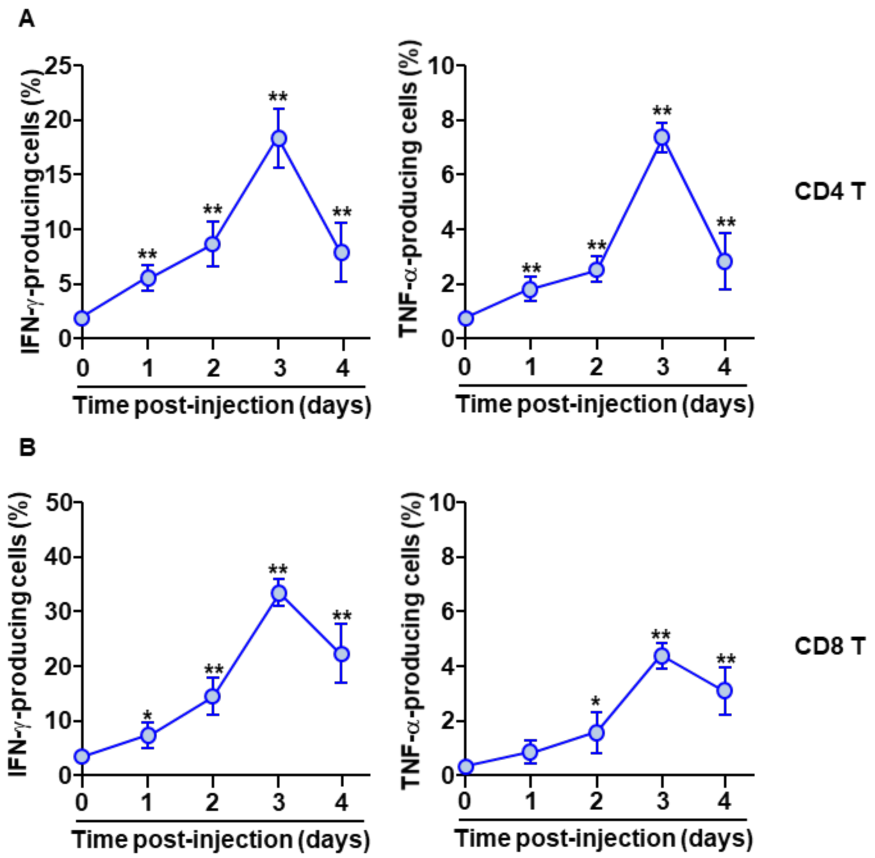

Figure S4. CFPs induce activation of T cells. CFP (50 mg/kg) was *i.n.* administered to C57BL/6 mice twice at 3 days interval. Time-dependent production of IFN-γ and TNF-α as measured by flow cytometry.
